# Uses of Physiologic Therapy

**Published:** 1913-10

**Authors:** Theodore Toepel

**Affiliations:** Atlanta, Ga.


### USES OF PHYSIOLOGIC THERAPY.

THEODORE TOEPEL, M. D., ATLANTA, GA.

The general principles that should govern the selection of physiologic measures may fairly be stated thus:

When a physiologic measure will produce the result desired, it should be used in preference to any drug—thus, the drip sheet or wet pack and massage rather than hypnotic drugs; cold water rather than antipyretic drugs; massage and electricity preferably to the constant use of laxatives.

When the result to be expected from a physiologic procedure is of slow development, drugs may be used meanwhile if needed, but should be discarded as soon as possible—thus, digitalis and nitroglycerine in some cases of heart disease submitted to Nanheim treatment, and palliative medication in cases of pulmonary tuberculosis treated physiologically. There is, however, no therapeutic or practical objection to the continued use of drugs auxiliary to physiologic treatment, provided that such medication is useful and necessary.

When equally efficacious measures not involving the use of apparatus or long journeys should be preferred—thus, baths at home over those at a spring or in an institution; respiratory exercises over pneumotherapy; massage over electricity.

When simple apparatus will suffice. elaborate apparatus is to be avoided—thus, gasometers are to be used rather than pneumatic cabinets. Exceptions may be made, however, when legitimate and suggestive effect is to be obtained from the more elaborate appliance.

When measures requiring elaborate apparatus or long journeys are, however, the ones needed or distinctly most efficient, they should be used without hesitation. Thus, an ocean voyage, a course at Nanheim, a sojourn at an altitude, or in a sanatorium, systematic hydrotherapy, baths, or inhalations of condensed and rarefied air, exposure to Roentgen—rays—may be the means of saving life, or hastening recovery, or of greatly promoting comfort, especially in chronic pulmonary, cardiac, metabolic, nervous and cutaneous affections, and should not be neglected.

In acute diseases, rest, hydrotherapy, thermotherapy, aerotherapy, dietetics, serotherapy offer the greater number of available measures. In convalescence from acute diseases, climate, light, massage, exercise are added to the list. In chronic affections, diet, exercise, hydrotherapy, thermotherapy, electrotherapy, massage, aerotherapy, organotherapy, suggestion, are the most important.

In affections of the digestive tract and of nutrition, diet, water, mineral waters, exercise, massage, light, electricity; radiotherapy, organotherapy; in affections of the circulatory systems, rest and exercise, baths, heat and cold, diet, pneumotherapy; in affections of the respiratory tract, pneumotherapy, inhalation, exercise, diet, hydrotherapy, light, radiotherapy; in affections of the nervous system, rest, hydrotherapy, electricity, exercise, massage, suggestion; in affections of the skeleton, rest, organotherapy, diet, manipulations, massage; in affections of the muscles, rest, exercise, massage, electricity; in affections of the excretory organs, water, massage, exercise electricity, saline infusions, thermotherapy, organotherapy—fulfil the major indications.

For stimulation, radiotherapy, electricity, exercise, massage, thermotherapy, hydrotherapy, organotherapy, pneumotherapy, climate, suggestion are employed. For sedation, rest, suggestion, massage, electricity, hydrotherapy thermotherapy, are useful.

The great extent of the field of physiologic therapeutics is thus evident. Physicians who begin to apply its measures never abandon them, as so many drugs are abandoned, but rather enlarge their use. It is gratifying to believe that this system has contributed materially to such enlargement.

